# Time Evolution of Plasmonic Features in Pentagonal Ag Clusters

**DOI:** 10.3390/molecules28155671

**Published:** 2023-07-26

**Authors:** Nicola Domenis, Pablo Grobas Illobre, Margherita Marsili, Mauro Stener, Daniele Toffoli, Emanuele Coccia

**Affiliations:** 1 Dipartimento di Scienze Chimiche e Farmaceutiche, Università degli Studi di Trieste, Via L Giorgieri 1, 34127 Trieste, Italy; 2 Scuola Normale Superiore, Piazza dei Cavalieri 7, 56126 Pisa, Italy; 3 Dipartimento di Fisica e Astronomia “Augusto Righi”, University of Bologna, Viale Berti Pichat 6/2, 40127 Bologna, Italy

**Keywords:** time-dependent density functional theory, transition contribution map, induced density

## Abstract

In the present work, we apply recently developed real-time descriptors to study the time evolution of plasmonic features of pentagonal Ag clusters. The method is based on the propagation of the time-dependent Schrödinger equation within a singly excited TDDFT ansatz. We use transition contribution maps (TCMs) and induced density to characterize the optical longitudinal and transverse response of such clusters, when interacting with pulses resonant with the low-energy (around 2–3 eV, A${}_{1}$) size-dependent or the high-energy (around 4 eV, E${}_{1}$) size-independent peak. TCMs plots on the analyzed clusters, Ag${}_{25}^{+}$ and Ag${}_{43}^{+}$ show off-diagonal peaks consistent with a plasmonic response when a longitudinal pulse resonant at A${}_{1}$ frequency is applied, and dominant diagonal spots, typical of a molecular transition, when a transverse E${}_{1}$ pulse is employed. Induced densities confirm this behavior, with a dipole-like charge distribution in the first case. The optical features show a time delay with respect to the evolution of the external pulse, consistent with those found in the literature for real-time TDDFT calculations on metal clusters.

## 1. Introduction

The optical response of metallic nanostructures to electromagnetic fields has been the subject of intense research activity for many years both at the experimental and theoretical level [1,2,3,4]. Such systems are characterized by the simultaneous presence of nearly free electrons confined to a nanometer-size scale and of intense plasmonic excitations of a collective nature. Such intense absorption bands, called Localized Surface Plasmon Resonance (LSPR) are easily tunable [5,6,7], by playing with size, shape, morphology, and chemical composition when nanoalloys are considered. Such versatility has made them suitable for a wide range of possible scientific and technological applications, ranging from optoelectronics to cancer treatment [1,2,3,8,9,10,11]. From a theoretical point of view, the plasmonic response of large particles (with size beyond 10 nm in each direction) can be predicted by employing variations of the original Mie theory [12] and classical electrodynamics models [13,14], but the extension of these approaches to smaller clusters is questionable due to the emergence of electronic confinement effects, which require quantum mechanical approaches to be properly described. In terms of rigorous information first-principles atomistic approaches, in particular, those based on Time-Dependent Density-Functional-Theory (TDDFT) [15] can make a decisive contribution. Regarding the systems of interest, the optical properties of noble metal nanoclusters with sizes up to some hundreds of atoms were widely studied. For example, tetrahedral Ag nanoclusters in the size range from 10 up to 120 atoms were investigated [16], and it was found that the spectra evolve from molecular-like to plasmon-like behavior as the size increases, in agreement with classical electrodynamics models. Moreover, the plasmon frequency decreases when the size of the cluster increases [16]. In analogous studies on Au clusters, it was found that the position of the peak in the absorption spectrum was weakly dependent on the shape of the cluster but was essentially related to its size and to the density functional theory (DFT) methodology, i.e., the exchange-correlation (xc-)functional used in the calculations [17]. Similar studies have been performed on Ag clusters of various sizes and shapes (octahedral, truncated octahedral, and icosahedral), confirming the red-shift of the peak position with cluster size and identifying the effect of the cluster shape on the absorption. In particular, a blue-shift with respect to increasing the number of facets is usually predicted by calculations [18]. Moreover, nanorods have also been considered, which are convenient because it is possible to reach a relatively large size in one dimension, thus exploring plasmonic behavior still keeping the cluster nuclearity relatively small [19,20,21,22]. A silver nanocage of 60 atoms was considered in Ref. [23], and compared with a hollow nanowire system, finding a similar behavior between the two systems, except that the nanowire gives rise to absorption peaks slightly red-shifted with respect to the nanocage. Larger silver nanocages were considered in [24], while in [25] gold and silver clusters up to M${}_{309}$ were considered at the TDDFT level of theory. Some recent applications of linear-response TDDFT considered tetrahedral Ag clusters up to 120 atoms [26], while the plasmonic response of silver, gold, and bimetallic Au–Ag particles of different shapes and sizes were considered in Ref. [27]. In particular, in these latter studies, it is shown how a tight-binding approximation either in the form of TD-DFTB [26] (time-dependent density functional tight binding) or in the linear-response calculation (TD-DFT+TB [27]) can lower by several orders of magnitude the CPU time requirements compared to DFT.

With “molecular plasmonics”, two scenarios are usually indicated: i) how the presence of a nanoparticle affects the electronic and optical properties of a molecule close to it [27,28,29,30,31,32,33,34,35,36]; ii) collective electronic response to a pulse in subnanometric systems, like metal clusters or molecular aggregates [21,37,38,39,40,41,42,43,44]. In this work, we focus on the latter definition of molecular plasmonics, looking at the time evolution of (collective) optical response of pentagonal silver clusters [19].

The most popular TDDFT approach used to obtain insights into the dynamics of the underlying mechanisms of the plasmonic response of nanoparticles is time propagation in the Kohn–Sham space (RT-RDDFT) [45,46,47,48,49,50,51]. Perhaps two of the most promising developments of RT-RDDFT to describe plasmonic systems are when combined with an efficient DFTB scheme [52,53] (RT-TDDFTB) or within orbital-free DFT [54,55]. Alternatively, the time-dependent Schrödinger Equation (TDSE) is propagated in the space of linear-response TDDFT eigenstates [56].

Several works have been dedicated to developing tools to analyze the electron density and dynamics affected by an external perturbation [49,57,58,59,60,61,62,63,64,65,66], also to extract possible plasmonic features [46,47,56,67,68].

**Figure 1:**
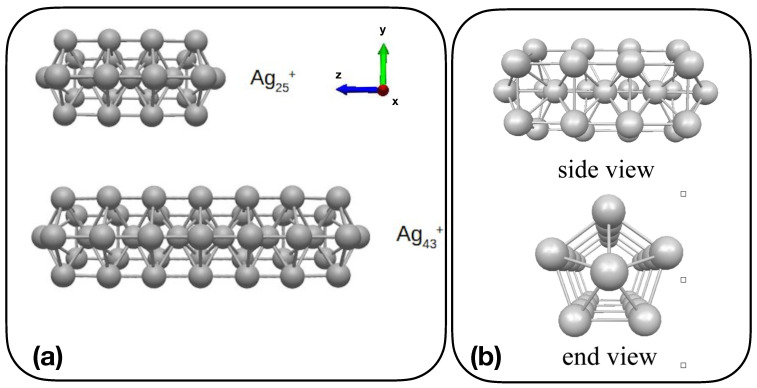
(**a**) Sketch representation of Ag${}_{25}^{+}$ and Ag${}_{43}^{+}$; (**b**) side and end view of Ag${}_{25}^{+}$.

In this paper, we present results from TDSE propagation [31,69,70,71] for Ag${}_{25}^{+}$ and Ag${}_{43}^{+}$ clusters (Figure 1), based on DFT and TDDFT representation of electronic ground and excited states. Electron dynamics are analyzed in terms of possible plasmonic features by means of time-resolved TCMs and induced density [56], with specific attention to longitudinal, i.e., along *z*, and transverse, i.e., *x* or *y*, excitations. The above-mentioned clusters show plasmonic behavior as a function of their length [19], and therefore are good candidates to address plasmonics with a feasible quantum approach.

The article is organized as follows: In Section 2, the theoretical framework is reviewed. Computational details are given in Section 3, results are presented and discussed in Section 4, and conclusions are provided in Section 5.

## 2. Theory

### 2.1. Real-Time Propagation

TDSE for the electronic dynamics is given by (bra-ket notation and atomic units are used here):

(1) $$i\frac{d}{dt}|\psi \left(t\right)\rangle =\hat{H}\left(t\right)|\psi \left(t\right)\rangle$$

where |ψ(t)〉 is the time-dependent wavefunction and $\hat{H}\left(t\right)$ is the time-dependent Hamiltonian, which includes the field-free Hamiltonian ${\hat{H}}_{el}$ and the interaction between the system dipole operator $\hat{\mu }$ with the external electromagnetic field F(t)

(2) $$\hat{H}\left(t\right)={\hat{H}}_{el}-\hat{\mu }\cdot F\left(t\right).$$

We have employed a Gaussian envelope function for the time-dependent external field:

(3) $$F\left(t\right)=F_{max}\exp\left(-\frac{{\left(t-t_{0}\right)}^{2}}{2\sigma^{2}}\right)\sin\left(\omega t\right),$$

where $F_{max}$ is the field amplitude (the intensity *I* is equal to $\frac{1}{2}{\left|F_{max}\right|}^{2}$), $t_{0}$ and σ are the center and the amplitude of the Gaussian, respectively, and ω is the pulse frequency.

In order to simulate a kick pulse, the external electric field F(t) is given by a narrow Gaussian function.

The electronic time-dependent wavefunction is expanded over the number $N_{\mathrm{states}}$ of eigenstates (in our case, the DFT ground state plus the $N_{\mathrm{states}}$ TDDFT eigenstates) as

(4) $$|\psi \left(t\right)\rangle =\sum_{m=0}^{N_{\mathrm{states}}}C_{m}\left(t\right)|\Phi_{m}\rangle .$$

In the expansion of Equation (4), $C_{m}\left(t\right)$ are time-dependent expansion coefficients, and $|\Phi_{m}\rangle$ represents the *m*-th time-independent TDDFT eigenstate, in the singly excited ansatz [56] of the system, with eigenvalue $E_{m}$.

The α-th component of time-dependent dipole moment is computed as follows [56]

(5) $$\mu_{\alpha }\left(t\right)=\sum_{l,m}C_{l}^{*}\left(t\right)C_{m}\left(t\right)\langle \Phi_{l}|{\hat{\mu }}_{\alpha }|\Phi_{m}\rangle .$$

The transition dipole $\langle \Phi_{l}|{\hat{\mu }}_{\alpha }|\Phi_{m}\rangle$ in Equation (5) is obtained from quantum-chemistry calculations [56].

### 2.2. Electron Dynamics Descriptors

#### 2.2.1. Time-Dependent TCM

TCM, originally defined in the frequency-domain in Ref. [72] is extended to the time domain [56] by

(6) TCM(ϵocc,ϵvir,t)=2∑i,aRe〈ψ(t)|P^ia|ψ(t)〉Gia(ϵocc,ϵvir),

where $G_{ia}\left(\epsilon_{occ},\epsilon_{vir}\right)$ is a Gaussian function used for convolution, and the projector ${\hat{P}}_{i}^{a}$ extracts the Slater determinant $|\Phi_{i}^{a}\rangle$ from the *m*-th electronic state:

(7) $$\begin{array}{ccc} |\Phi_{m}\rangle & = & \sum_{i,a}d_{i,m}^{a}|\Phi_{i}^{a}\rangle \end{array}$$

(8) $$\begin{array}{ccc} {\hat{P}}_{i}^{a}|\Phi_{m}\rangle & = & d_{i,m}^{a}|\Phi_{i}^{a}\rangle , \end{array}$$

with *i* and *a* indicating occupied and virtual MOs, respectively.

Using the expansion in Equation (4), and with $\langle \Phi_{l}|{\hat{P}}_{i}^{a}|\Phi_{m}\rangle ={\left(d_{i,l}^{a}\right)}^{*}d_{i,m}^{a}$, one obtains

(9) TCM(ϵocc,ϵvir,t)=2∑i,a∑l,mReCl*(t)Cm(t)(di,la)*di,maGia(ϵocc,ϵvir).

#### 2.2.2. Time-Dependent Induced Density

The transition density associated with the *m*-th electronic transition is defined in its diagonal form as

(10) $$\Gamma_{m}\left(r\right)=\langle \Phi_{m}|\hat{\rho }\left(r,r\right)|\Phi_{0}\rangle ,$$

where $\hat{\rho }\left(r,r\right)$ is the diagonal reduced one-electron density matrix operator, and $|\Phi_{0}\rangle$ is the DFT ground-state Slater determinant. r is the collective electronic coordinate.

From the time-dependent expectation value of $\hat{\rho }\left(r,r\right)$ one obtains the time-dependent induced density Γ(r,r,t) [56]

(11) $$\Gamma \left(r,r,t\right)=\Gamma_{1}\left(r,r,t\right)+\Gamma_{2}\left(r,r,t\right)$$

with

(12) Γ1(r,r,t)=2∑m>0Re[Cm(t)]〈Φ0|ρ^(r,r)|Φm〉

(13) =2∑m>0Re[Cm(t)]Γm(r,r)

and

(14) $$\Gamma_{2}\left(r,r,t\right)=\sum_{l>0,m>0}C_{l}^{*}\left(t\right)C_{m}\left(t\right)\langle \Phi_{l}|\hat{\rho }\left(r,r\right)|\Phi_{m}\rangle .$$

$\Gamma_{2}\left(r,r,t\right)$ can be neglected since a small intensity, which would lead to a non-appreciable excited-state population, is assumed.

## 3. Computational Details

Ground-state geometry coordinates of Ag clusters have been taken from Ref. [19]. A +1 charge characterizes the clusters, in order to use a closed-shell formulation of DFT and TDDFT. TDDFT calculations have been performed using AMS suite for codes [73], with a DZ basis set with relativistic corrections and frozen core up to 4*p*. Standard Casida approach via Davidson diagonalization has been employed. PBE [74] functional has been chosen.

Real-time propagation has been carried out with WaveT [32,56]. WaveT simulations used as ingredients eigenenergies and transition dipole moments from AMS calculations [56,75]. In order to fill the visible energy range up to 5 eV, we included 750 and 2100 electronic states for Ag${}_{25}^{+1}$ and Ag${}_{43}^{+1}$, respectively. 100-fs electron dynamics were simulated, with a time step of 1.21 as. A pulse intensity of $5\times 10^{8}$ W/cm${}^{2}$ was employed. FWHM of the resonant pulse from Equation (3) is equal to 15 fs, and that of the kick pulse is equal to 47 as. $t_{0}$ was set to 36 fs.

The induced density resulting from a pulse resonant with the frequency of the longitudinal plasmonic peak was represented using an isovalue of 3 $\times \;10^{-5}$. The analog analysis for the transverse plasmonic peak involved an isovalue of 10${}^{-5}$ and 3 × 10${}^{-5}$ for the Ag${}_{25}^{+}$ and Ag${}_{43}^{+}$ structures, respectively.

## 4. Results and Discussion

**Figure 2:**
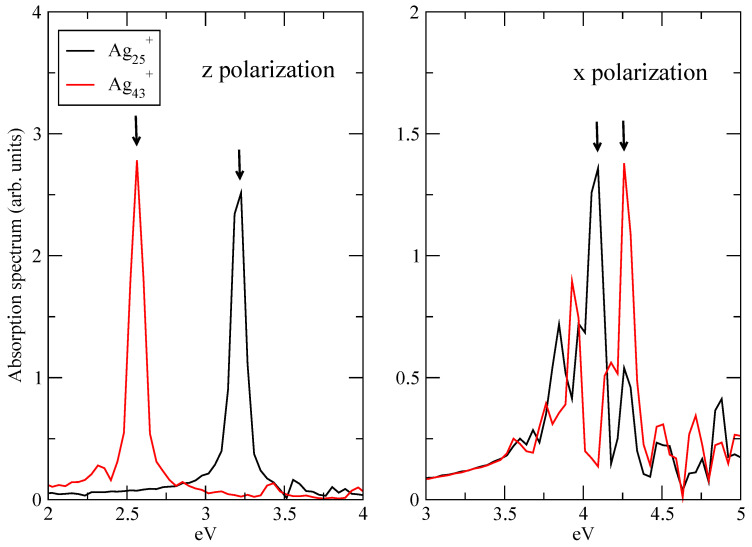
Absorption spectra A${}_{1}$ (*z* polarization, left panel) and E${}_{1}$ (*x* polarization, right panel) peaks of Ag${}_{25}^{+}$ and Ag${}_{43}^{+}$. Spectra computed using a kick pulse. Spectra normalized to unity. Arrows indicate peaks corresponding to the selected frequencies for the plasmon analysis.

Analysis of plasmonic effects in the electron dynamics of Ag${}_{25}^{+}$ and Ag${}_{43}^{+}$ has been performed by focusing on the longitudinal, i.e., *z*, response of A${}_{1}$ transition and on the transverse, i.e., *x* or *y*, response of E${}_{1}$ transition. Because of the symmetry of the clusters (Figure 1), *x* and *y* optical responses are equivalent. According to Ref. [19] A${}_{1}$ transition is the “plasmonic” one, which changes with the size of the cluster, while E${}_{1}$ transition is the molecular-like one, which does not show a dependence on the size of the system in terms of frequency. A${}_{1}$ and E${}_{1}$ frequencies have been obtained by inspecting the longitudinal and transverse components of the absorption spectrum, computed using the kick pulse. Figure 2 reports such absorption spectra, for Ag${}_{25}^{+}$ and Ag${}_{43}^{+}$. Spectra have been computed as the Fourier transform of the time-dependent dipole moment of Equation (5). From the longitudinal response (left panel of Figure 2) one extracts a maximum frequency, which corresponds to the A${}_{1}$ transition in Ag${}_{25}^{+}$ and Ag${}_{43}^{+}$: ω=3.326 eV for Ag${}_{25}^{+}$ and ω=2.565 eV for Ag${}_{43}^{+}$. E${}_{1}$ frequencies are instead taken from the maxima of the transverse spectra in the right panel of Figure 2: ω=4.078 eV for Ag${}_{25}^{+}$ and ω=4.258 eV for Ag${}_{43}^{+}$. Arrows in Figure 2 are a guide for the eye.

**Figure 3:**
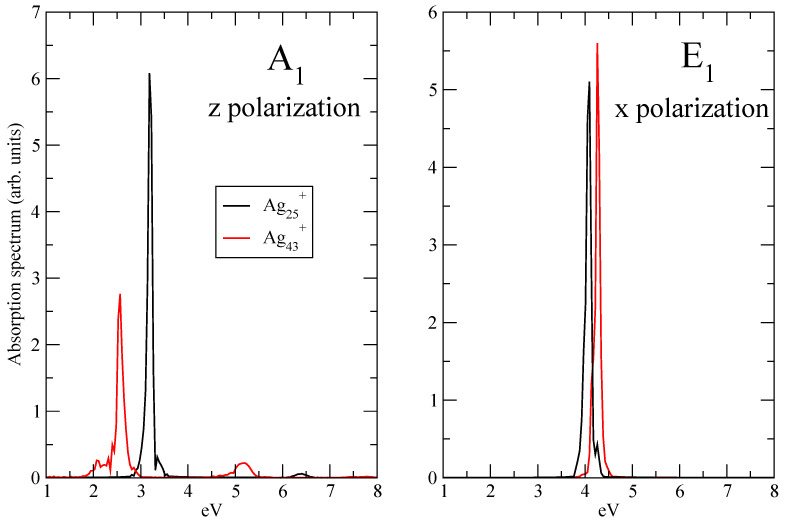
Absorption spectra A${}_{1}$ (*z* polarization, left panel) and E${}_{1}$ (*x* polarization, right panel) peaks of Ag${}_{25}^{+}$ and Ag${}_{43}^{+}$. Spectra computed using the pulse of Equation (3). Spectra normalized to unity.

Let the panel of Figure 3 show the Fourier transform of the *z* component of the time-dependent dipole moment of Ag${}_{25}^{+}$ and Ag${}_{43}^{+}$ (see Equation (5)) when the pulse frequency is resonant with A${}_{1}$ transition. The Fourier transform of the *x* component of the time-dependent dipole moment of Ag${}_{25}^{+}$ and Ag${}_{43}^{+}$ (see Equation (5)) with the pulse frequency equal to the E${}_{1}$ transition is reported in the right panel of Figure 3. In all the cases, a main and narrow peak is located at the transition frequency, as expected. No other transition is involved, only a low signal for the longitudinal spectrum of Ag${}_{43}^{+}$ is found at around 5.1 eV: these findings suggest that the electron dynamics are dominated by the photoinduced excitation. In other words, the time-resolved features observed by means of TCM and induced density, as shown below, are directly attributable to A${}_{1}$ and E${}_{1}$ transitions.

Analysis of the electron dynamics of Ag${}_{25}^{+}$ and Ag${}_{43}^{+}$ in terms of TCM and induced density has been accomplished considering, for the figures collected below, four time snapshots: 27.6, 36, 44.4, and 60 fs, which correspond to an increasing pulse, pulse maximum, decrease of the pulse, and zero pulse, respectively. These snapshots have been extracted from the whole TDSE dynamics.

**Figure 4:**
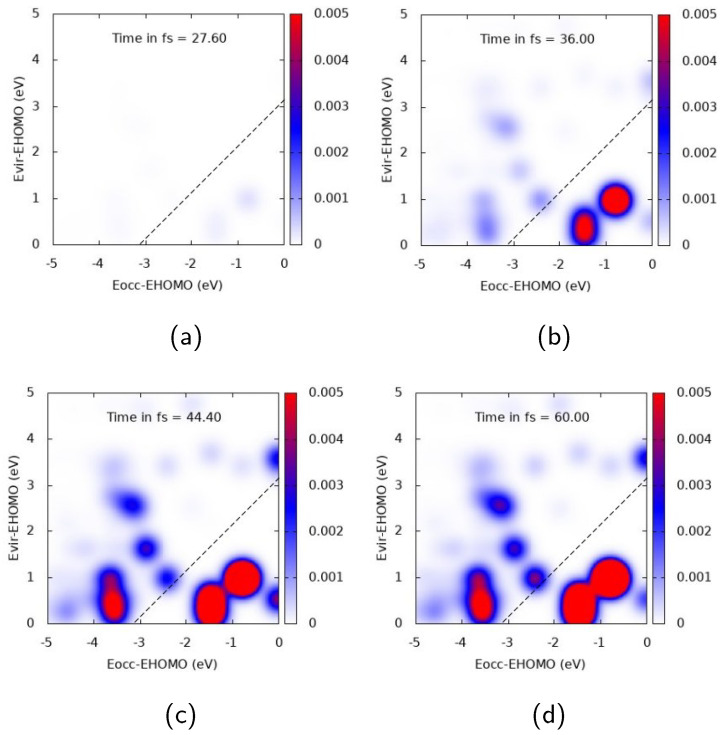
TCM plots for Ag${}_{25}^{+}$ at the A${}_{1}$ peak (ω=3.326 eV), longitudinal *z* component. (**a**) Snapshot at 27.6 fs; (**b**) Snapshot at 36.0 fs; (**c**) Snapshot at 44.4 fs; (**d**) Snapshot at 60.0 fs.

**Figure 5:**
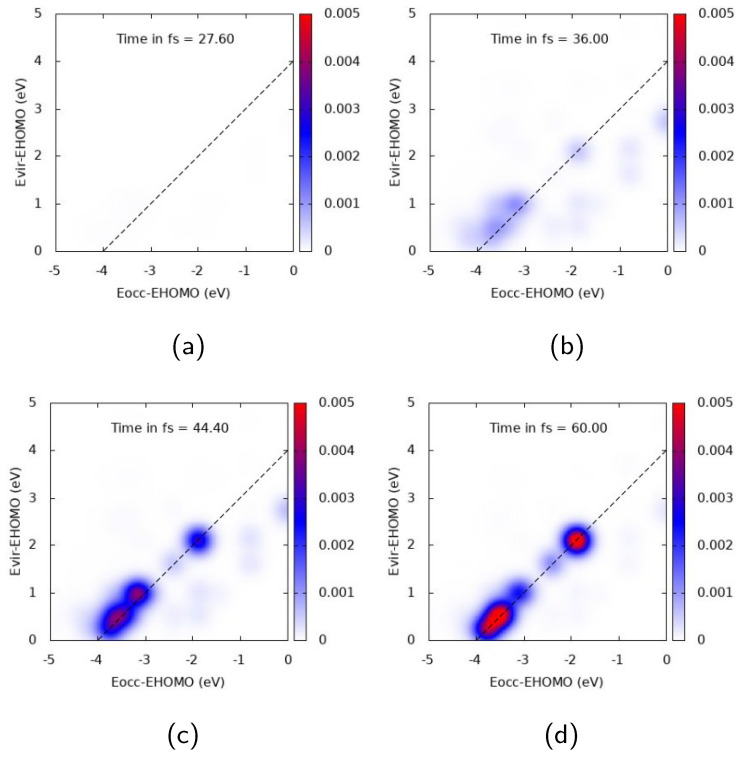
TCM plots for Ag${}_{25}^{+}$ at the E${}_{1}$ peak (ω=4.078 eV), transverse components. (**a**) Snapshot at 27.6 fs; (**b**) Snapshot at 36.0 fs; (**c**) Snapshot at 44.4 fs; (**d**) Snapshot at 60.0 fs.

Figure 4 and Figure 5 report TCM plots for Ag${}_{25}^{+}$. TCM is a two-dimensional descriptor able to detect collective features, looking at the single-electron orbital occupation in time: the appearance of off-diagonal spots, with the diagonal corresponding to the pulse frequency, indicates a plasmon-like excitation [39,72,76]. TCM spots are defined with respect to the energies of virtual and occupied MOs, scaled with HOMO energy. When the cluster interacts with a pulse polarized longitudinally with a central frequency corresponding to the A${}_{1}$ transition, i.e., ω=3.326 eV, a clear collective response is observed, as reported in Figure 4. Several off-diagonal spots appear, below and above the diagonal. In the first case, the most intense and large ones already appear at 36 fs, and correspond to a one-electron transition from inner MOs 1-2 eV below the HOMO energy to a large portion of virtual MOs. In the second one, a strong spot appears at 44.4 fs, indicating electron promotion from even deeper MOs at around 3.5 eV below the HOMO energy. A rather delayed response of the system seems to occur, since the maximum intensity of the most extended TCM signal (above and below the diagonal) is found after the pulse maximum (top right panel of Figure 4).

The nature of the electronic excitation of Ag${}_{25}^{+}$ dramatically changes when the pulse is polarized transversely to the cluster and resonant with the E${}_{1}$ transition (ω = 4.078 eV), as shown in Figure 5. In this case, a strong diagonal structure is found, with the highest intensity at 44.4 and 60 fs, giving evidence of the fact that the photoinduced electronic density is dominated by a molecule-like excitation. At 60 fs, the largest spots describe a transition from deep MOs which are 3.5–4 eV below the HOMO to low-energy virtual MOs, and a transition from inner MOs (around 2 eV below the HOMO) to virtual ones 2 eV above the HOMO. TCM spots reach their maximum value well beyond the pulse maximum, confirming that the optical response of the cluster is characterized by a temporal delay with respect to the evolution of the pulse.

**Figure 6:**
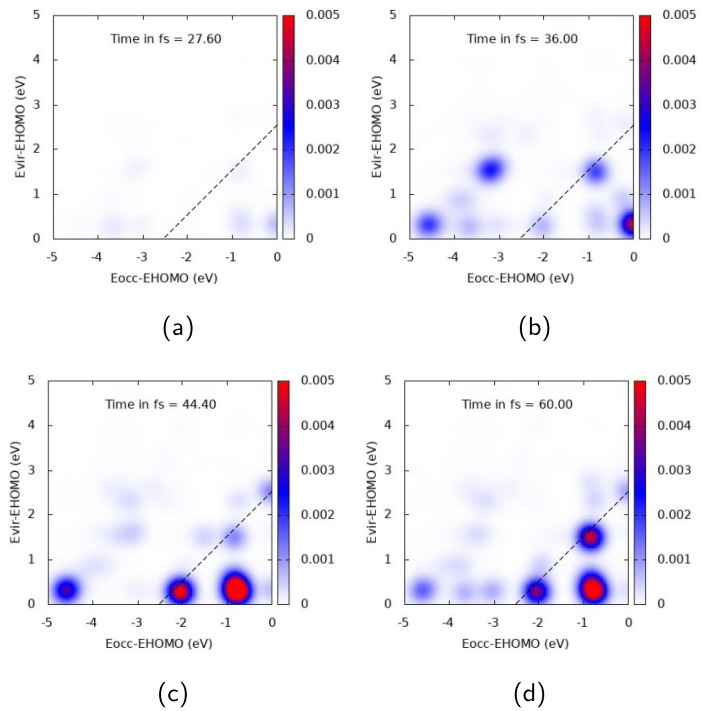
TCM plots for Ag${}_{43}^{+}$ at the A${}_{1}$ peak (ω=2.565 eV), longitudinal *z* component. (**a**) Snapshot at 27.6 fs; (**b**) Snapshot at 36.0 fs; (**c**) Snapshot at 44.4 fs; (**d**) Snapshot at 60.0 fs.

**Figure 7:**
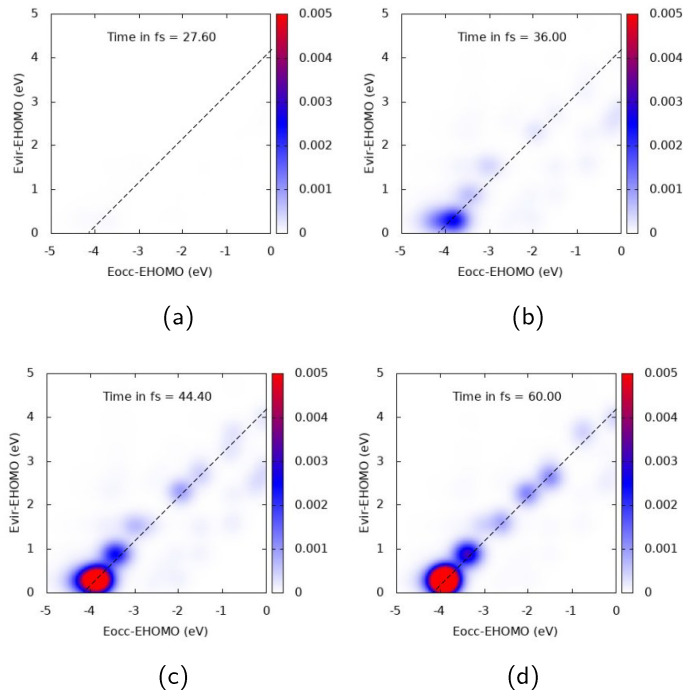
TCM plots for Ag${}_{43}^{+}$ at the “E${}_{1}$” peak (ω=4.258 eV), transverse components. (**a**) Snapshot at 27.6 fs; (**b**) Snapshot at 36.0 fs; (**c**) Snapshot at 44.4 fs; (**d**) Snapshot at 60.0 fs.

Similar general comments can be applied to Ag${}_{43}^{+}$ TCM plots, reported in Figure 6 and Figure 7 for A${}_{1}$ longitudinal and E${}_{1}$ transverse responses, respectively. In Figure 6, a strong off-diagonal spot appears at 44.4 fs. The spot describes a one-electron transition from inner occupied MOs to virtual MOs just above the HOMO. At 44.4 and 60 fs an oscillation of the two diagonal spots is also seen, which can be interpreted as a coherent time-dependent superposition of one-electron transitions. Several diagonal peaks instead characterize the TCM in Figure 7, with the most intense one describing an electron promotion from deep MOs (around 4 eV below the HOMO energy) to MOs just above the HOMO energy. The delayed plasmon-like response is also observed for Ag${}_{43}^{+}$: intensity of off-diagonal peaks obtains its maximum well beyond the pulse maximum, i.e., 36 fs (see Figure 6. Similar behavior is also found for the molecular-like response of Figure 7.

It is worth mentioning that coherent electron dynamics have been carried out, thus no signal decay, due to dephasing or relaxation channels, which can be observed in this framework.

**Figure 8:**
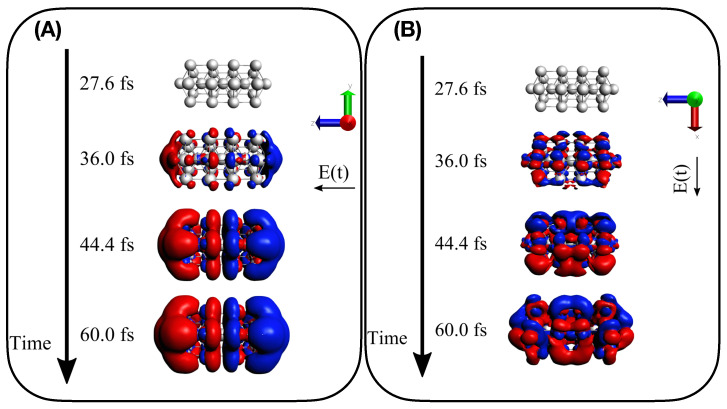
Induced density $\Gamma_{1}\left(r,r,t\right)$ as computed in Equation (13) for four different snapshots of electron dynamics of Ag${}_{25}^{+}$: (**A**) for A${}_{1}$ longitudinal transition and (**B**) for E${}_{1}$ transverse transition.

**Figure 9:**
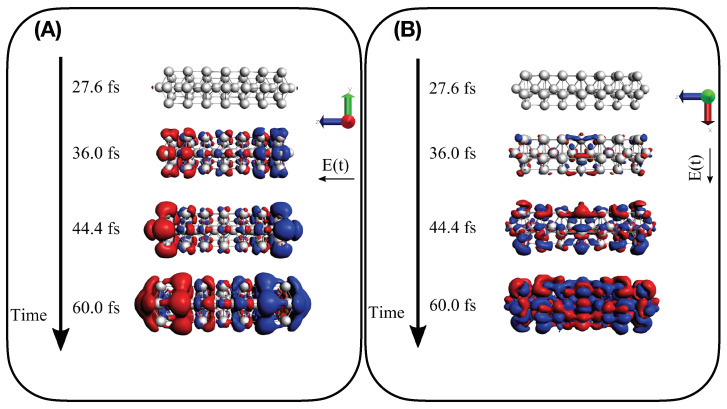
Induced density $\Gamma_{1}\left(r,r,t\right)$ as computed in Equation (13) for four different snapshots of electron dynamics of Ag${}_{43}^{+}$: (**A**) for A${}_{1}$ longitudinal transition and (**B**) for E${}_{1}$ transverse transition.

Findings given by TCM plots are further supported by the analysis of the time-dependent induced density of Equation (13). Panels (A) and (B) of Figure 8 report $\Gamma_{1}\left(r,r,t\right)$ for Ag${}_{25}^{+}$ interacting with a longitudinal A${}_{1}$-resonant and transverse E${}_{1}$ transition, respectively. A dipole-like density is observed in the presence of the A${}_{1}$-centered pulse, typical of a collective response, whereas a more irregular density is found when the pulse with E${}_{1}$ central frequency is applied, which corresponds to a standard molecular-like excitation with no plasmonic behavior. Time-dependent induced density for the two pulses has been also computed for Ag${}_{43}^{+}$, as reported in Figure 9. Similar behavior is found, even though the dipole-like density of Ag${}_{43}^{+}$ in panel A) of Figure 9 appears more “fragmented” than the Ag${}_{25}^{+}$. In panel A) of Figure 9 we substantially observe the delayed photoinduced generation of the plasmon. No plasmon decay is observed because coherent dynamics have been performed, as already mentioned.

Concerning the time delay of the optical response of the Ag clusters with respect to the pulse, similarly to our findings, a delayed response of the electronic system with respect to the incoming pulse has been reported within the real-time TDDFT (RT-TDDFT) dynamics of an Ag${}_{55}$ cluster, for which both dipole moment and charge density difference are at their maxima when the external electric field is already faded out [77]. Interestingly, in the same paper, it was shown how the electron-electron interaction, provided by the TDDFT kernel, is responsible for such delayed features which are absent in an independent particle propagation. This confirms how the electron-electron interaction, at least the part of it that stems from the approximated xc-kernel, is embedded in the present electronic dynamics of Ag${}_{25}^{+}$ and Ag${}_{43}^{+}$.

An electronic population dynamics well beyond the pulse duration was also found in RT-TDDFT calculations of water molecules on an Ag/Pt cluster [78] and of a small Ag cluster, for which off-diagonal elements of the density matrix in the space of MOs kept rising [79]. Transition contribution maps of the light-induced RT-TDDFT electronic dynamics of an Ag${}_{561}$ cluster, instead, showed only diagonal contributions after the pulse duration [47].

Linear dephasing time experiments found long-lived components, with dephasing times up to ∼30 fs for ∼90 nm Ag nanoclusters [80]. Indeed future work will be dedicated to the time-evolution of plasmon features in the presence of electron dephasing and relaxation channels, in order to study how the environment, i.e., what is not electronic degrees of freedom of the system, interacts with the excitations [32,81,82,83].

## 5. Conclusions

In this work, TDSE was applied to study plasmonic features of pentagonal silver clusters Ag${}_{25}^{+}$ and Ag${}_{43}^{+}$.

TDSE was coupled with a linear-response TDDFT description of electronic excited states and with a suite of post-processing tools, able to extract one-electron properties from the time-dependent electronic wavefunction. TDDFT results were obtained using the AMS package, while electronic dynamics were performed via WaveT code.

According to the investigated spectral region, we observed collective features of the electronic excitation or a purely “molecular” response: low-energy transitions are seen to be characterized by off-diagonal peaks in TCM plots, whereas only diagonal spots (i.e., with a single-electron energy difference corresponding to the pulse frequency) are found for high-energy transitions of the absorption spectrum. These features were confirmed by the time-dependent induced densities.

Moreover, an analysis of various snapshots of the time propagation shows that the collective response of the system is generally retarded with respect to the incoming pulse: regardless of the size of the Ag cluster, the largest intensity in the TCM signal is seen beyond the pulse maximum, i.e., 36 fs.

The present results show how the time-resolved theoretical approach is able to capture the physics of metal clusters and, possibly, of composite systems. Future applications of time-resolved TCM and induced density will regard, e.g., the study of the electron dynamics in QM/continuum simulations, as performed for plasmon-enhanced photocatalysis [84,85,86,87], and understanding of the interplay between plasmons and quantum coherence [81,82,83].

## Data Availability

The data that support the findings of this study are available from the corresponding author upon reasonable request.
